# Metastatic non-small cell lung cancer presenting with an orbital metastasis: a case report

**DOI:** 10.1186/1757-1626-1-89

**Published:** 2008-08-13

**Authors:** Arun Azad

**Affiliations:** 1 Ludwig Institute for Cancer Research, University of Melbourne, Austin Hospital, Austin Health, 145-163 Studley Road, Heidelberg, Victoria, 3084, Australia

## Abstract

Metastatic disease to the orbit occurs in up to 7% of cancers. In approximately 20% of cases, there is no diagnosis of cancer at the time of presentation with orbital metastatic disease. This is a case of a 53-year-old female smoker whose initial presentation of metastatic non-small cell lung cancer was with an orbital metastasis.

## Case presentation

A 53-year-old Caucasian woman presented with a one month history of a painful right eye associated with decreased vision and diplopia. Her only other symptom was right knee pain. Past medical history was unremarkable and she took no medications. She had smoked 20 cigarettes per day for approximately 35 years. She drank alcohol only occasionally. She worked as a receptionist, and had no children. There was no family history of malignancy.

On examination, she was a well-looking woman. Height was 165 cm and weight 60 kg. Vital signs were within normal limits. Examination of the right eye revealed conjunctival injection, proptosis, markedly reduced visual acuity, and restricted extra-ocular movements (particularly lateral gaze). The rest of the physical examination was unremarkable, apart from mild tenderness over the distal right femur.

Computed tomography (CT) of the brain demonstrated an enhancing 2.1 × 1.6 cm right orbital mass. Histology from a fine needle aspiration biopsy (FNAB) was consistent with a moderately differentiated, mucin-producing adenocarcinoma. On immunohistochemistry, cytokeratin 7 (CK7) was positive; however cytokeratin 20 (CK20), carcinoembryonic antigen (CEA), estrogen and progesterone receptors were all negative. Interestingly, thyroid transcription factor-1 was also negative, which is unusual for primary lung adenocarcinoma [1].

**Figure 1:**
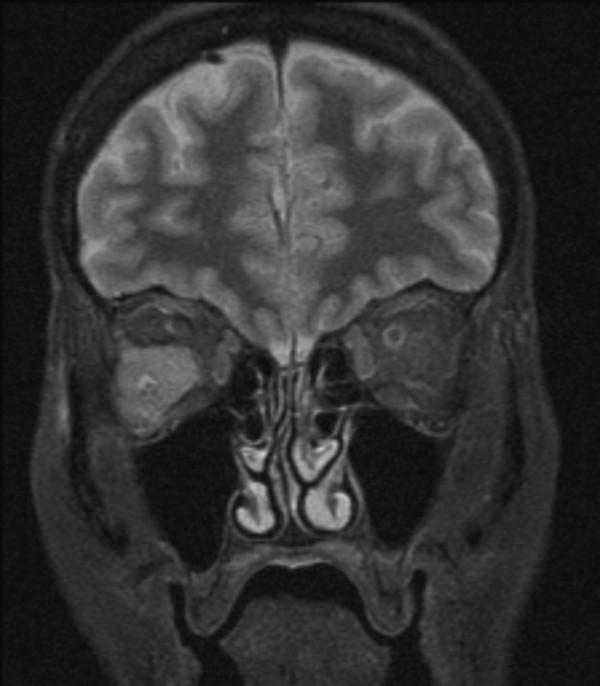
**Coronal T1 MRI brain and orbit**. MRI performed 2 weeks after fine needle aspiration biopsy of the right orbital mass, but prior to commencing chemotherapy. The right orbital mass is shown causing displacement of the optic nerve.

After referral to medical oncology, further investigations were performed. Tumour markers (CEA, CA-125, CA 15.3) were normal. CT of the chest and abdomen revealed a spiculated 1.7 cm lesion in the left lower lobe and a 2.3 cm left adrenal mass. Whole body bone scan showed widespread osteoblastic bone metastases, including the distal right femur. Magnetic resonance imaging (MRI) of the brain demonstrated the right orbital mass which was abutting the right lateral rectus muscle and displacing the optic nerve (see Figure 1).

Despite TTF-1 being negative, the diagnosis of metastatic adenocarcinoma of the lung was made due to the history of smoking and radiological pattern of disease. Given the burden of systemic disease, the patient was commenced on chemotherapy with a view to administering radiotherapy to the right eye at a later date. Carboplatin (area under the curve 5; day 1) and vinorelbine (25 mg/m^2^, day 1 and day 8) were given in 3 weekly cycles. At the time of writing, the patient had received three cycles of treatment. She had stable disease on restaging and no significant toxicity.

Metastatic disease to the eye most commonly involves the choroid [2]. By comparison, metastatic disease to the orbit is relatively uncommon, occurring in up to 7% of all cancers [3]. Metastases to the orbit also account for less than 5% of all orbital tumours [4]. The commonest primary tumour sites for orbital metastases include breast, prostate and lung [5]. In approximately 20% of cases, orbital metastases are the initial manifestation of systemic malignancy [6]; this is more common with lung rather than breast cancers [7].

Compared with CT, MRI provides superior resolution of both orbital and intracerebral metastases, which are not uncommon in the setting of an orbital metastasis [8]. The diagnosis can be confirmed with FNAB or at the time of excision. FNAB is diagnostic in greater than 90% of cases [9].

Local control is achieved with surgery or radiotherapy; however in the setting of significant systemic disease, local control may need to be deferred to allow administration of systemic treatment. The typical dose of radiotherapy is 30–50 Gy, with up to 90% of patients experiencing symptomatic improvement [10]. However, most patients also require systemic treatment. The prognosis of patients with metastatic disease to the orbit is poor. Median survival is typically just over one year, although breast carcinoma patients have slightly better outcomes [6,7].

## Competing interests

The author declares that they have no competing interests.

## Consent

Written informed consent was obtained from the patient for publication of this case report and accompanying images. A copy of the written consent is available for review by the Editor-in-Chief of this journal.

## References

[B1] Stenhouse G, Fyfe N, King G, Chapman A, Kerr KM (2004). Thyroid transcription factor 1 in pulmonary adenocarcinoma. J Clin Pathol.

[B2] De Potter P (1998). Ocular manifestations of cancer. Curr Opin Ophthalmol.

[B3] Macedo J, Machado M, Araujo A, Angelico V, Lopes J (2007). Orbital metastasis as a rare form of clinical presentation of non-small cell lung cancer. J Thor Oncol.

[B4] Mena A, Pardo J (2002). Orbital metastasis as the initial manifestation of small cell lung cancer. Acta Ophthalmol.

[B5] Wolstencroft SJ, Hodder SC, Askill CF, Sugar AW, Jones EW, Griffiths AP (1999). Orbital metastasis due to interval lobular carcinoma of the breast. Arch Ophthalmol.

[B6] Shields JA, Shields CL, Brotman HK, Carvalho C, Perez N, Eagle RC (2001). Cancer metastatic to the orbit: the 2000 Robert M. Curtis Lecture. Ophthal Plast Reconstr Surg.

[B7] Char DH, Miller T, Kroll S (1997). Orbital metastases: diagnosis and course. Br J Ophthalmol.

[B8] Ratanatharathorn V, Powers WE, Grimm J, Steverson N, Han I, Ahmad K, Lattin PB (1991). Eye metastasis from carcinoma of the breast: diagnosis, radiation treatment and results. Cancer Treat Rev.

[B9] Tijl J, Koornneef L (1991). Fine needle aspiration biopsy in orbital tumours. Br J Ophthalmol.

[B10] Glasburn JR, Klionsky M, Brady LW (1984). Radiation therapy for metastatic diseases involving the orbit. Am J Clin Oncol.

